# Differential Association of Free, Conjugated, and Bound Forms of Polyamines and Transcript Abundance of Their Biosynthetic and Catabolic Genes During Drought/Salinity Stress in Tomato (*Solanum lycopersicum* L.) Leaves

**DOI:** 10.3389/fpls.2021.743568

**Published:** 2021-10-15

**Authors:** Rakesh K. Upadhyay, Tahira Fatima, Avtar K. Handa, Autar K. Mattoo

**Affiliations:** ^1^Sustainable Agricultural Systems Laboratory, Henry A. Wallace Beltsville Agricultural Research Center, United States Department of Agriculture-Agricultural Research Service, Beltsville, MD, United States; ^2^Center of Plant Biology, Department of Horticulture and Landscape Architecture, Purdue University, West Lafayette, IN, United States

**Keywords:** polyamine, putrescine (PUT), spermidine (SPD), spermine (SPM), tomato, drought, salt

## Abstract

Polyamines have been implicated in ameliorating the detrimental effects of drought and saline conditions on plant growth and development. The independent impact of these two abiotic stresses on polyamine (PA) biosynthesis, catabolism, and homeostasis, as well as on their transcript abundance in tomato leaves, is presented here. We show that the total levels of putrescine (PUT), spermidine (SPD), and spermine (SPM) increase up to 72 h during drought and up to 48 h during salinity stress before their precipitable drop thereafter. Thus, tomato plants maintain survivability to drought as well as salinity stress for up to 3 and 2 days, respectively. Independent multivariant analyses of drought and salinity stress kinetic data separately showed a closer association with levels of free, conjugated, and bound forms of SPD and SPM, but not with free or bound PUT. However, combined multivariant analyses showed a closer association of free SPD, conjugated SPD, and bound SPD with both stresses; SPD-bound and SPM conjugated with drought; and free SPM and conjugated PUT with salinity stress, respectively. PA biosynthesis genes, *ARG1*, *SPDS1*, and *SAMDc3*, segregated with drought and *SPDS2* with salinity stress. PA catabolic genes *CuAO4-like* and *PAO4* were associated with drought and salinity stresses, respectively, suggesting differential involvement of PA biosynthesis and catabolic genes in drought and salinity stresses. Pearson correlation indicated mostly positive correlations between the levels of free, conjugated, and bound forms of PUT, SPD, and SPM under drought and salinity stress. However, negative correlations were mostly seen between the levels of various forms of the PAs and their biosynthesis/catabolic genes. Levels of different PA forms had a twofold higher negative correlation during drought as compared to salinity stress (66 vs. 32) and with transcript levels of PA biosynthesis and catabolic genes. Transcripts of light-harvesting chlorophyll a/b-binding genes were generally positively associated with different forms of PAs but negatively to carbon flow genes. Most of the PA biosynthesis genes were coordinately regulated under both stresses. Collectively, these results indicate that PAs are distinctly regulated under drought and salinity stress with different but specific homologs of PA biosynthesis and catabolic genes contributing to the accumulation of free, conjugated, and bound forms of PAs.

## Introduction

Abiotic environmental factors, such as drought and salinity, are significant plant stressors with a major impact on plant development and productivity, thus, causing serious agricultural yield losses (Flowers, 2004; Godfray et al., 2010; Tester and Langridge, 2010; Golldack et al., 2014). The complex regulatory processes of plant adaptation to drought and salt involve control of water flux and cellular osmotic adjustment *via* biosynthesis of osmoprotectants (Hasegawa et al., 2000; Flowers, 2004; Munns, 2005; Ashraf and Akram, 2009). Additionally, drought and salinity have major detrimental impacts on the cellular energy supply and redox homeostasis that are balanced by global reprogramming of plant primary metabolism and altering cellular architecture (Chen et al., 2005; Baena-González et al., 2007; Jaspers and Kangasjärvi, 2010; Miller et al., 2010; Zhu et al., 2010). One important class of cellular metabolites acting as osmoreceptors is polyamines (PAs). PAs as osmo-protectants have been shown to protect plants against adverse environmental conditions (Alcázar et al., 2010; Mattoo et al., 2015b; Gong et al., 2016; Handa et al., 2018), notably against drought and salinity in various crops and crop models (Capell et al., 2004; Cona et al., 2006; Kusano et al., 2008; Alcázar et al., 2010; Minocha et al., 2014; Mattoo et al., 2015b).

Polyamines are found either as free, conjugated, bound, or non-covalently conjugated (NCC) forms in nature [reviewed by Chen et al. (2019), and references there in]. Free PAs covalently bound with biomacromolecules, such as proteins, nucleic acids, uronic acids, or lignin by ionic and hydrogen bonds, are known as bound PAs (Chen et al., 2019). In the physiological pH range, free-PAs are fully protonated and positively charged, and can electrostatically combine with negatively charged biomacromolecules (acidic proteins, membrane phospholipids, and nucleic acids) in the organism and known as conjugated PAs (Igarashi and Kashiwagi, 2015; Chen et al., 2019). The conjugated PAs have a wide range of biological functions in plant growth and development. They have been associated with the regulation of enzyme activity, DNA replication, gene transcription, cell division, and membrane stability (Chen et al., 2019). However, little is known about the association of different polyamine forms with abiotic stresses, particularly in tomatoes.

Polyamines (SPM, SPD, and PUT) were shown to regulate the stomata pore opening and closing to control water loss from plants during drought conditions (Liu et al., 2000). In alfalfa, PUT treatment was shown to improve seed germination and increase the growth indexes under polyethylene glycol (PEG 4000)-mediated drought stress both *in vitro* and in a pot experiment (Zeid and Shedeed, 2006). Arabidopsis mutant *acl5*/*Spm*, which lacks SPM, is hypersensitive to high salt and drought and was rescued by SPM pretreatment but not by PUT or SPD, suggesting that the drought hypersensitivity of the mutant is due to SPM deficiency (Yamaguchi et al., 2007). *Arabidopsis* ADC2 deletion mutant was found extremely sensitive to salt stress, which was alleviated by applying exogenous PUT (Naka et al., 2010). Furthermore, SPM was found strongly associated with drought resistance in apple (Liu et al., 2010) and cherry tomatoes (Montesinos-Pereira et al., 2014). Moreover, SPD and SPM relieved the inhibitory effects of drought stress and promoted grain filling and drought resistance in wheat, while PUT had the opposite effect (Liu et al., 2016). The application of different types and varying concentrations of exogenous PAs was shown to alleviate the effects of NaCl stress in *Brassica juncea* and strawberry seedlings (Verma and Mishra, 2005; Li et al., 2010). Similarly, the application of SPM and SPD were found to result in increased reactive oxygen metabolism and photosynthesis, which, in turn, improved plant growth and reduced the inhibitory effects of salt stress (Meng et al., 2015; Baniasadi et al., 2018). Interestingly, recent information has indicated that SPD and thermospermine (TSPM) share molecular functions related to quality control pathways for tightly regulated mRNAs at the level of translation [Poidevin et al. (2019) and references therein].

The plant polyamine metabolic pathway is mediated by several enzymes in the sequential synthesis of PUT, SPD, and SPM (Mattoo et al., 2015a). Arginine (Arg) decarboxylase (ADC; EC 4.1.1.19) converts Arg to PUT while, alternatively, Ornithine (Orn) decarboxylase (ODC; EC 4.1.1.7) converts Orn to PUT (Mattoo et al., 2015a; Tsaniklidis et al., 2016). Arginase (ARG; EC 3.5.3.1) balances both pathways by converting Arg to Orn. Higher PAs, SPD, and SPM are synthesized from PUT by spermidine synthase (SPDS; EC 2.5.1.16) and spermine synthase (SPMS; EC 2.5.1.22), respectively. Decarboxylation of SAM by SAM-decarboxylase (SAMDc; EC 4.1.1.50) adds amino propyl groups in a sequential manner to PUT and SPD for the synthesis of SPD and SPM, respectively. A sequentially back conversion of PAs from SPM to SPD to PUT under specific conditions/processes is achieved by PA oxidase (PAO; EC 1.5.3.11) and copper-containing amine oxidase (CuAO; EC 1.4.3.6). Such interconversion of PAs is thought to contribute to the tight regulation of PAs homeostasis (Møller and McPherson, 1998; Moschou et al., 2012; Tavladoraki et al., 2016), which results in the production of the signaling molecule H_2_O_2_ as a byproduct (Sebela et al., 2001; Cona et al., 2003, 2006; Angelini et al., 2008; Moschou et al., 2008). Differential activation of PA metabolic pathway genes in tomato leaves under heat and cold stress conditions has also been determined (Upadhyay et al., 2020).

Novel approaches that utilized genetic engineering to accumulate PAs in agronomic and model plants, including tobacco, rice, tomato, Arabidopsis, pear, and potato, and overexpressed SAM decarboxylase were found to be tolerant against given stresses, such as salt, osmotic, and heat (Wi et al., 2006; Alcázar et al., 2010), while overexpression of spermidine synthase led to tolerance against drought, salt, and oxidative stresses (Kasukabe et al., 2004, 2006; He et al., 2008; Wen et al., 2008; Alcázar et al., 2010; Neily et al., 2011). Similarly, transgenic *Lotus tenuis* plants expressing *ADC* adjusted better to osmotic adjustment (5.8-fold) under salinity stress (Espasandin et al., 2018).

A plant leaf is a photosynthetically active aerial tissue mainly responsible for carbon assimilation activities and is highly prone to environmental changes, which in turn, affect the yield and productivity of the whole plant. How PA biosynthesis/catabolism pathways are modulated in tomato leaf tissue in response to drought or salinity stress and how they coordinate with specific changes in PA levels in their various forms (free, conjugated, and bound) are yet to be fully determined. Here, we present genetic data involving the quantification of 18 tomato gene transcripts involved in PA biosynthesis and catabolism in relation to drought and salt stress of leaves, and expression dynamics of other 22 genes that include nuclear-encoded photosynthetic and plastid-encoded protein genes, carbon fixation-encoding genes, together with stress-specific marker genes to establish correlative indices with PA abundance. The results obtained outline specific gene flow responses to conditions of drought and salt stress and influence differential PA abundance.

## Materials and Methods

### Plant Growth Conditions

Wild-type tomato (*Solanum lycopersicum* cv. Ailsa Craig) plants were grown in a temperature-controlled (22°C ± 3) greenhouse under natural light conditions in metro-mix 360 soil (sun-grow). After transplanting, plants were grown for 4 weeks (28 days) before initiating stress treatments. All the harvested samples were immediately frozen in liquid nitrogen and stored at −80°C until used.

### Drought and Salt Stress Treatments

The transplanted tomato plants were grown in soil for 28 days and used for stress experiments. Salt and drought treatments were given in a temperature-controlled (22°C ± 3) greenhouse as described previously (Upadhyay et al., 2019). Briefly, drought treatment involved withholding watering of plants for 7 days (168 h). Leaf samples were collected at 0, 24 (day 1), 48 (day 2), 72 (day 3), and 168 h (day 7) after initiating the drought treatment. Control samples were also collected at indicated time points. Salt (200-mM NaCl) was used for salinity stress. The plants were daily irrigated with 200 ml of NaCl (200 mM) solution. Leaf samples of salt-treated plants were collected at 0, 2, 6, 48, and 96 h, along with control samples.

### Selection and Extraction of Gene Sequences Encoding for Polyamine Metabolism in Tomato

Gene expression of tomato PA metabolic pathway enzymes was carried out (listed in Supplementary Table S1). Briefly, for PUT accumulation, two *arginase*-*encoding genes* (*SlARG1, 2*); two *arginine decarboxylase-encoding genes* (*SlADC1, 2*); *agmatine iminohydrolase*/*deiminase* (*SlAIH*); *N-carbamoyl putrescine amidase* (*SlCPA*); and two *ornithine decarboxylase-encoding genes* (*SlODC1, 2*) were selected; for SPD and SPM accumulation, three *S-adenosylmethionine decarboxylase-encoding genes* (*SlSAMDc1, 2, and 3*); two *spermidine synthase-*encoding genes (*SlSPDS1, 2*); and one *spermine synthase-*encoding (*SPMS*) gene were chosen for transcript analysis. Concurrently, four catabolic genes, including two *flavin-dependent polyamine oxidases* (*SlPAO4-like* and *SlPAO2*), and two *copper-dependent amine oxidases* (*SlCuAO4* and *SlCuAO-like*) were chosen to study PA catabolism along with biosynthesis (Supplementary Table S1). To study the flow of ornithine toward the proline metabolic pathway, a single copy ornithine aminotransferase-encoding gene (*SlOAT1)* was chosen to address the PA pathway shift toward the proline pathway in drought and salinity conditions (Upadhyay et al., 2020).

### Specific Marker Genes Selected for Drought/Salt Stress

For drought stress responses, eight genes, namely, *SlDREB1* (Solyc06g050520) and *SlDREB2* (Solyc05g052410); *SlNCED1* (Solyc07g056570) and *SlNCED2* (Solyc08g016720); *SlRD29A* (Solyc03g025810) and *SlDELLA* (Solyc11g011260) genes; *SlWIRKY57* (Solyc05g012500) and *SlJUB1* (Solyc05g021090) served as drought marker genes. Three salt responsive genes, *SlSOS1* (Solyc01g005020), *SlNHX3* (Solyc01g067710), and *SlNHX4* (Solyc01g098190) were used as salt-stress-marker genes. Sequences were extracted from the tomato genome database [International Tomato Genome Sequencing Consortium (SGN^1^) as Arabidopsis homologs].

### Photosynthesis Processes and Carbon Fixation Pathway-Encoding Genes

The effect of stress on the carbon assimilation of the plant was tested using known genes that encode proteins involved in photosynthesis, such as the nuclear-encoded light-harvesting chlorophyll a/b-binding protein genes [*SlLhcb1* (Solyc03g005770.1), *SlLhcb2* (Solyc12g006140.1), *SlLhcb3* (Solyc07g063600.2), *SlLhcb4* (Solyc09g014520.2), *SlLhcb5* (Solyc06g063370.2), and *SlLhcb6* (Solyc01g105050.2)], as well as plastid-encoded photosystem II (PSII) protein genes *SlpsbA* and *SlaccD*. Tomato *SlpsbA* gene [encoding the D1 protein of PSII (NC_007898.3)] and *accD* gene (encoding the beta-carboxyl transferase subunit of acetyl-CoA carboxylase) information were extracted from tomato chloroplast genome (NC_007898.3). *SlLhcb1-6* genes, *phosphoenolpyruvate carboxylase* (*SlPEPC*) (AJ243417.1/Solyc07g062530.2) and *isocitrate dehydrogenase* (*SlICDH*) (XM_010314428.2/Solyc11g011930.1.1) genes were used as markers for the carbon flow during drought/salt stress (Supplementary Table S2).

### RNA Extraction, cDNA Preparation, and Quantitative PCR Analysis

Frozen tomato leaf tissue was ground to a fine powder (100 mg) to extract total RNA using Plant RNeasy kit (Qiagen, Hilden, Germany). DNase (Qiagen) was used to remove genomic DNA, followed by a cleanup with a RNeasy mini kit (Qiagen). RNA quality was checked by spectrophotometer, and the RNAs with an *A_260__/__280_* with ratios of 1.8–2 were subjected to agarose gel electrophoresis to ensure the presence of intact rRNA band before their selection for cDNA preparation (Upadhyay and Mattoo, 2018). RNA (2 μg) was used for cDNA synthesis using an iScriptadvanced cDNA synthesis kit (Bio-Rad, Hercules, CA, United States), followed by a 10-fold dilution for further use. Quantitative real-time PCR (qRT-PCR) was performed using Sso Advanced Universal SYBR Green Supermix (Bio-Rad) in a Bio-Rad cycler (CFX96 Bio-Rad Real-Time PCR machine). PCR conditions were sequentially 95°C for 5 min, 95°C for 15 s, and 60°C for 60 s (40 cycles), followed by melt curve analysis (Shukla et al., 2017). Gene expression was quantified using the ΔΔC_T_ method (Livak and Schmittgen, 2001). Two reference genes (*SlTIP41* and *SlUBI3)* were used for normalizing the expression of the target genes (Expósito-Rodríguez et al., 2008; Mascia et al., 2010). MIQE (Minimum Information for Publication of Quantitative real-time PCR Experiments) guidelines were followed for quantification of genes (Bustin et al., 2009). Relative fold changes were calculated as previously described (Shukla et al., 2017). Primer sequences for 42 genes used for qRT-PCR are listed in Supplementary Table S2. Relative qRT-PCR data represent average ± SD from a minimum of three independent biological replicates.

### Quantification of Polyamines—Putrescine, Spermidine, and Spermine—by High-Pressure-Liquid Chromatography

Freeze-dried tomato leaf material was extracted and dansylated as described previously (Torrigiani et al., 2012; Anwar et al., 2019) with some modifications. About 50 mg of each finely ground leaf sample was suspended in 800 μl of 5% ice-cold perchloric acid (PCA) and homogenized in a handheld homogenizer. After centrifugation at 20,000 g for 30 min at 4°C, the supernatant (free and conjugated PAs) and residue pellet (bound PAs) were collected separately. The 20,000-*g* pellet was washed two times with 5% PCA and resuspended in 800 μl of 5% cold PCA. An aliquot (0.5 ml) of supernatant and suspended pellet was hydrolyzed in an equal volume of 6-N HCl for 18 h at 110°C to release conjugated and bound insoluble PAs, respectively. About 100-μl aliquots of soluble supernatant before and after hydrolysis, and of the suspended pellet were then quantified (soluble, conjugated, and bound PAs, respectively). To each aliquot, saturated sodium carbonate (200 μl) and 1,7-heptanediamine (400 μl, as an internal standard) were added and then dansylated with dansyl chloride for 60 min at 60°C in the dark. Dansylation was terminated by adding 100-μl proline and incubating the reaction mixture for 30 min at 60°C. Other details were the same as described earlier (Anwar et al., 2019). For PAs recovery and calibration curves, authentic PA standards (Sigma-Aldrich, St. Louis, MO, United States) were used as control. PAs were integrated and quantified using Millennium 4.0 Chromatography Manager software from Waters Corporation. Samples of PCA-soluble, PCA-soluble hydrolyzed with HCl, and PCA-insoluble after hydrolysis were quantified, and are designated as free, free + conjugated, and bound forms of each PA, respectively (Anwar et al., 2019).

### Statistical, Principal Component, and Cystoscope Analysis

Data were examined with Graph-Pad Prism (version 8.2.1) statistical software package, and two-way ANOVA (a mixed model) with recommended Geisser–Greenhouse Correction was performed for deriving statistical significance. Multiple comparisons against “oh” within data points were performed using Tukey/Dunnett test. Differences among treatments were considered significant at *p* < 0.05. PCA, correlation, and Cytoscape analyses were performed using XLSTAT, Excel, and Cytoscape (Shannon et al., 2003) programs, respectively. The correlation coefficients were determined using the Microsoft EXCEL program and analyzed using the Expression Correlation App of Cytoscape program (Shannon et al., 2003). All values were normalized as% of the initial value (0-time point) before determining the correlation coefficient *r*. Gene abbreviations are the same as in Supplementary Tables S1, S2. Only correlations > 0.8 were plotted. All plotted correlations were significant at a *p*-value of 0.001.

## Results

### Drought Stress Effects on Polyamine Levels and Transcript Levels and Polyamine Metabolic and Photosynthesis Genes

#### Kinetics of Changes in Cellular Contents of Polyamines During Drought Stress in Tomato Leaves

**FIGURE 1:**
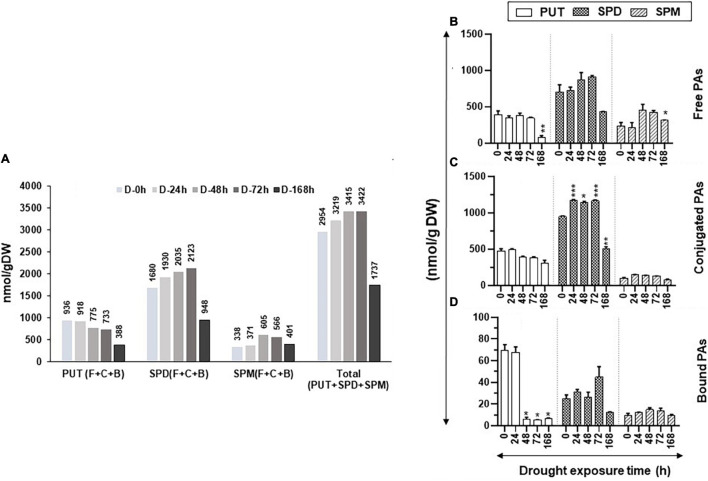
Changes in the levels of free, conjugated, and bound forms of putrescine (PUT), spermidine (SPD), and spermine (SPM) during drought stress in tomato leaves. The drought stress was imposed on tomato plants by withholding water. Leaf samples from a minimum of three plants were harvested at the indicated time periods and immediately frozen in liquid N_2_ and then stored at –80°C until analyzed. High-pressure liquid chromatography (HPLC) was used to determine the levels of free, bound, and conjugated forms of polyamines (PAs) as described in the Materials and Methods section. **(A)** Changes in the total amount of free, bound, and conjugated PUT, SPD, and SPM under drought stress; **(B)** free PUT, SPD, and SPM; **(C)** conjugated PUT, SPD, and SPM; **(D)** bound PUT, SPD, and SPM, respectively, during the increasing period of drought stress. Shown are mean + STE from three independent biological replicates. Statistical significance (*p*-value) was derived using the Graph prism pad program *via* Tukey test/*t*-test. *, **, *** indicate significance at *p*-value < 0.05, <0.005, and <0.0005, respectively.

Figure 1A shows the changes in total (Free + Conjugate + Bound) cellular content of PUT, SPD, and SPM and PAs during the increasing period of drought stress. The total cellular levels of PUT continued to decline with an increasing drought period, whereas the total cellular contents of SPD and SPM increased until 72 h of drought before showing precipitable declines after 168-h drought (Figure 1A). Collectively, these results indicate that the cellular metabolic activities of the PAs pathway continued up to at least 72 h after initiation of drought stress.

The increasing period of drought stress did not significantly change the levels of the free PUT (PUT-F), except after prolonged drought stress, when its levels precipitously dropped at 168 h of drought (Figure 1B). Similar trends were seen for the SPD-F as its level declined after 168 h of drought (Figure 1B). The levels of SPM-F, however, showed a significant increase after 48 and 72 h of drought before its decline at 168 h of drought (Figure 1B). The conjugated PUT (PUT-C) levels steadily declined throughout the experimental period, but these changes were not significantly different at all sampling periods (Figure 1C). The SPD-C levels, however, significantly increased after 24, 48, and 72 h of drought and declined after 168 h of withholding water (Figure 1C). The levels of SPM-C did not show noticeable changes during the experiment (Figure 1C). The levels of bound PUT (PUT-B) steeply declined after 24 h of drought (Figure 1D) and, by 48, 72, and 168 h, the levels were insignificant (Figure 1D). The small contribution of PUT-B levels to total PUT did not appreciably affect total PUT (Figure 1A). We interpret the sharp decline in PUT-B to be possibly due to the onset of drought stress, leading to the breakdown of PUT-binding macromolecules. The levels of SPD-B and SPM-B did not appreciably change during the increasing period of drought. Taken together, these results suggest that only prolonged drought stress affects the levels of PAs, especially PUT-F and PUT-B. A significant increase in SPD-C but not in SPD-F after 24–72 h of drought stress indicates that plants maintain homeostasis for SPD-F during the drought stress by conjugating SPD. The higher levels of SPM-F but not SPM-C suggest a limited role for SPM conjugation during drought stress. Statistical differences for polyamine abundances during drought stress kinetics are shown in Supplementary Table S3.

#### Kinetic Changes in the Gene Transcripts of Polyamines Biosynthesis and Catabolism

**FIGURE 2:**
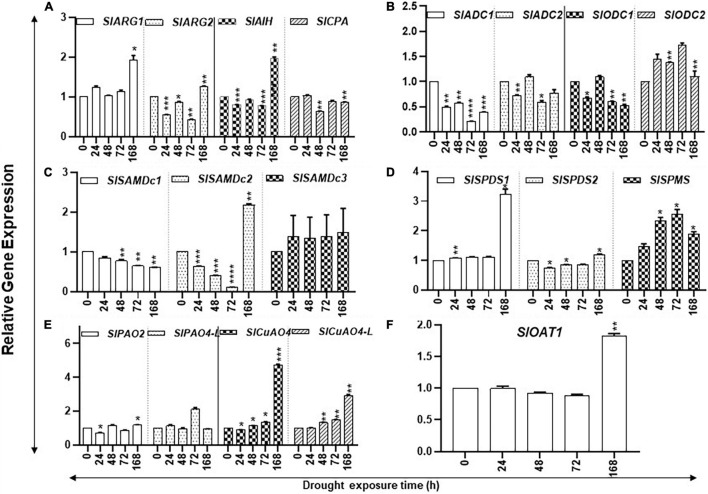
Kinetics of drought-induced expression of PA metabolic pathway genes in tomato leaves. Total RNAs were extracted from tomato leaves harvested after withholding water for 0, 24, 48, 72, and 168 h, respectively. The steady state levels of transcripts of various PAs biosynthesis and catabolism genes were determined by qRT-PCR as described in section “Materials and Methods.” Genes included were of PUT biosynthesis (*SlARG1, 2*, *SlADC1, 2 SlODC1, 2, SlAIH*, and *SlCPA*), SPD, and SPM biosynthesis (*SlSAMDc1, 2, 3, SlSPDS1, 2*, and *SlSPMS*); and catabolism (*SlPAO2* and *SlPAO4-Like; SlCuAO* and *SlCuAO4-like*) pathways, respectively. *SlUBI3* and *SlTIP41* were used as reference genes. Other details were the same as in Figure 1. **** indicates significance at *p*-value of <0.00005.

Prolonged drought stress affected the accumulation of transcript levels of several PAs biosynthesis pathway genes. The steady state levels of *SlARG1/2, SlAIH, SlSPDS1*, and *SlOAT1* transcripts increased significantly only after 168 h of drought stress (Figures 2A,D,F), whereas the steady state levels of *SlODC2* and *SlSPMS* transcripts significantly increased after 24 h, continuing until 72 h before declining at 168 h of drought (Figures 2B,D). Levels of *SlADC1*, *SlSAMdDc1/2*, and *SlODC1* steadily declined during the increasing period of drought stress (Figures 2B,C). Transcript levels of *SlOAT1 SlARG1, SlAIH, SlSAMdc2, SlSPDS1, SlCuAO4*, and *SlCuAO4-like* genes in PA metabolism had significant increases only after 168 h of drought. The molecular basis of their increase after 168 h is not clear. It is possible that these genes respond only upon prolonged drought, but this remains to be tested.

The PAs catabolism pathway involves catabolism of PUT by copper amine oxidases (CuAO4 and CuAO4-like) and of SPD/SPM by flavin adenine dinucleotide (FAD)-dependent PA oxidases (PAO2 and PAO4-like) (Cona et al., 2006; Angelini et al., 2008; Tavladoraki et al., 2016). Expression of *SlPAO2* remained low during the drought stress and significantly declined at 24 h, followed by an increase in 168-h samples (Figure 2E). The transcript levels of *SlPAO4-like* remained similar at most time points of kinetics, except for an increase after 72 h of drought. The steady state levels of *SlCuAO4* and *SlCuAO4-like* gene transcripts significantly increased during the experiment and continued to increase up to 168 h of water withholding (Figure 2E). We interpret these results to suggest that tomato leaves exhibit noticeable changes in PA biosynthesis and catabolism until 72 h but undergo dramatic changes after prolonged drought stress. This has ramifications in the recovery of plants from drought stress as it is likely that plants have the potential to recover within 72 h of drought while prolonged drought stress becomes irreversible.

#### Levels of the Chlorophyll a/b-Binding, Photosystem II, and Carbon Flow Genes During Drought

**FIGURE 3:**
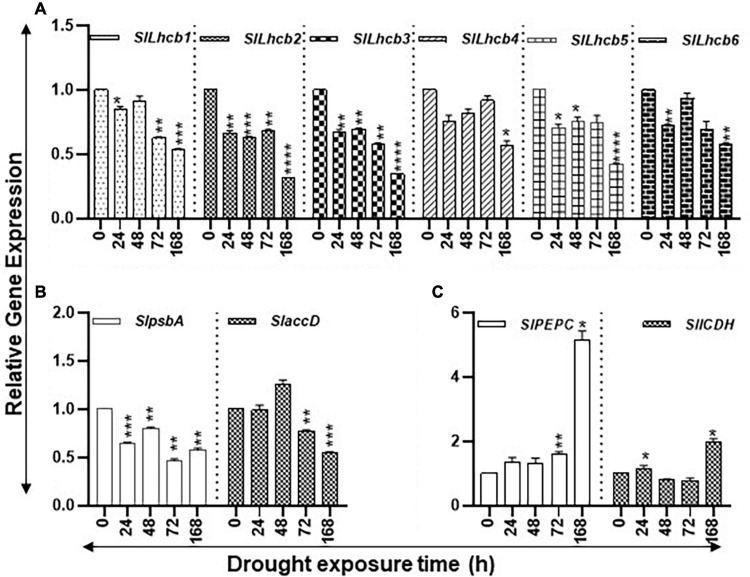
Effect of drought on the steady state abundance of chlorophyll a/b-binding, plastid-encoded photosystem II (PSII), and carbon flow gene transcripts in tomato leaf tissue. Quantitative PCR was used to determine the steady state levels of transcripts of **(A)** nuclear-encoded light-harvesting chlorophyll a/b-binding protein genes (*Lhcb1-6);* **(B)** Plastid-encoded photosystem II (PSII) genes (*psbA* and *accD)*; and **(C)** *phosphoenolpyruvate carboxylase* (*PEPC*) and *isocitrate dehydrogenase* (*ICDH*) genes. Other details are the same as in Figure 1. **** indicates significance at *p*-value of <0.00005.

The study state levels of carbon assimilation genes during the increasing period of drought stress are shown in Figure 3. The transcript levels of six nuclear-encoded light-harvesting chlorophyll a/b-binding genes (*SlLhcb1*, *SlLhcb2*, *SlLhcb3*, *SlLhcb4*, *SlLhcb5*, and *SlLhcb6*) steadily declined during the 168-h course of water withholding (Figure 3A). The levels of these genes significantly declined after 24 h of drought and continued to decline during the prolonged 168-h drought. The two plastid-encoded photosystem II (PSII) genes, *SlpsbA* and *SlaccD*, significantly declined at 168 h of drought compared to control of the 0-h time point but transiently increased in the 48-h samples (Figure 3B). Patterns of transcript levels of *SlPEPC* (*phosphoenolpyruvate carboxylase*) and *SlICDH (isocitrate dehydrogenase*) genes were analyzed as markers for the carbon flow in response to drought stress (Figure 3C). The transcript levels of *SlPEPC* were significantly higher at 72 h and greatly increased by 168 h while *SlICDH* transcript levels were significantly higher at 24 and 168 h with a dip in their levels at 24 and 72 h.

#### Expression of Stress-Responsive Genes and Transcription Factors Under Drought Stress

**FIGURE 4:**
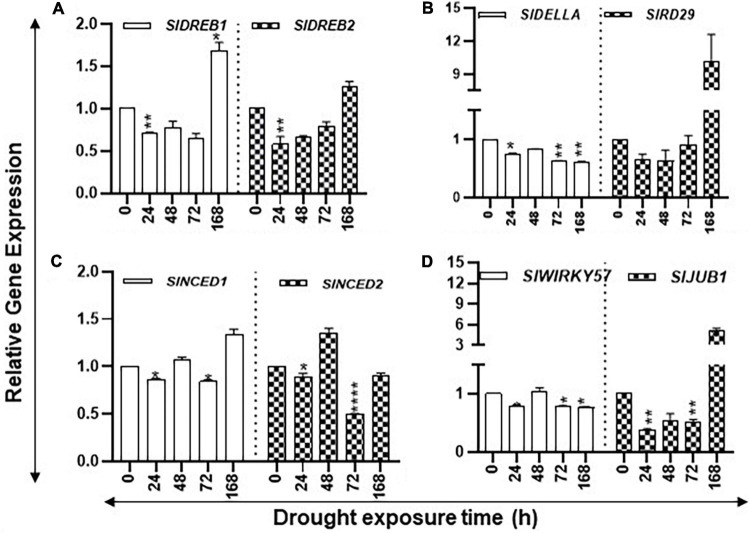
Changes in the expression levels of drought stress-related marker genes during the increasing period of drought in tomato leaves. Quantitative PCR was used to determine the steady state levels of transcripts of *SlDREB1/2, SlNCED1/2, SlRD29A, SlWIRKY57*, and *SlJUB1* genes. Other details are the same as in Figure 1.

Expression of desiccation-responsive *SlRD29A*, *SlDELLA*, *SlWIRKY57*, *SlJUB1*, *SlDREB1*, and *SlDREB2* genes, together with ABA biosynthesis pathway genes *SlNCED1* and *SlNCED2*, was determined since their induction in response to drought stress has been reported in the literature. After an initial decrease in the transcript levels of *SlDREB1/2*, *SlRD29*, *SlNCED1*, and *SlJUB1*, their steady state transcript levels were found to significantly increase after 168 h of drought (Figure 4). Transcript levels of *SlDELLA* and *SlWRKY57* declined during the increasing drought period, whereas mixed patterns of accumulation of *SlNCED2* transcripts were apparent during the stress period with their decline seen at 168 h (Figure 4). Taken together, these results indicate that seedlings perceive drought stress within 24 h that becomes severe upon prolonged drought.

### Salt Stress and Changes in Polyamines Levels, Metabolic-Transcripts, and Photosynthesis Genes

#### Kinetic Changes in the Content of Total Polyamines During Salt Stress in Tomato Leaves

**FIGURE 5:**
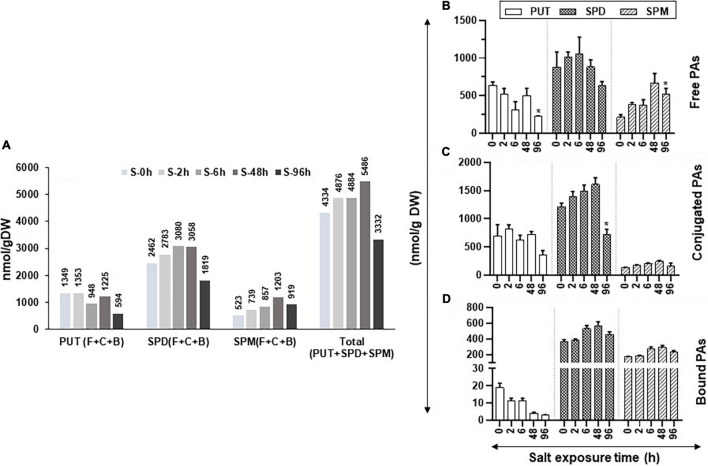
Changes in the levels of free, conjugated, and bound forms of PUT, SPD, and SPM in tomato leaves during salt stress. Salt stress was imposed on tomato plants by irrigating plants daily with 200-mM NaCl (salt stress). Leaf samples from a minimum of three plants were harvested at 0, 2, 6, 48, and 96 h and immediately frozen in liquid N_2_ and then stored at –80°C until analyzed. HPLC was used to determine the levels of free, bound, and conjugated forms of PAs. **(A)** Changes in the total amount of free, bound, and conjugated forms of PUT, SPD, and SPM; **(B)** free PUT, SPD, and SPM; **(C)** conjugated PUT, SPD, and SPM; **(D)** bound PUT, SPD, and SPM, respectively, during salt stress, respectively. Other details were the same as in Figure 1.

Changes in total (free + conjugated + bound) cellular content of PUT, SPD, and SPM during salinity stress are shown in Figure 5A. The total cellular levels of PUT continued to decline with the increasing saline stress period, whereas the total cellular contents of SPD and SPM increased until 48 h of saline stress before their precipitable decline after 168 h of stress (Figure 5A). The trend of total cellular PA content (all forms of PUT + SPD + SPM) followed a pattern similar to total SPD and SPM, likely since SPD was found to be the dominating cellular PA in tomato leaves under saline stress (Figure 5A). These results are similar to those obtained under drought stress, indicating that, during drought and salinity stresses, the cellular metabolic activities of the PAs pathway continue up to at least 72 h for drought and 48 h for salinity stress (Figures 1A, 5A).

During the increasing period of salt stress, the levels of free PUT (PUT-F) gradually decreased, except at 48-h treatment, while a significant decline occurred after 96 h of salt treatment (Figure 5B). Levels of SPD-F steadily increased until 6 h before declining thereafter until 96 h of salt treatment while SPM-F levels steadily increased until 48 h before their decline at 96 h of treatment (Figure 5B). The PUT-C levels showed mixed patterns with a precipitable decline observed after 96 h of salt treatment (Figure 5C). Levels of SPD-C increased up to 48-h salt-stress before their steep decline at 96 h (Figure 5C). In contrast, the SPM-C levels were low and remained similar throughout the salt-treatment period (Figure 5C).

The bound form of PUT (PUT-B) steadily declined throughout the salt treatment while both SPD-B and SPM-B levels increased after 6 h of salt treatment and remained higher than that at 0-h control up to 96 h of salt exposure (Figure 5D). These results indicate that exposure to salinity alters cellular levels of PAs, SPD, and SPM in particular. Not only did the salt treatment increase the cellular levels of SPD-F and SPM-F but also converted some of the SPD-F to SPD-C and SPD-B, likely to maintain the homeostasis of cellular SPD (Figures 5B–D). Taken together, these results suggest that both SPD and SPM play key roles in the salinity response of tomato leaves. Quantified levels of free, conjugated and bound PAs and their statistical analyses are shown in Supplementary Table S4.

#### Kinetic Changes in the Levels of Polyamine Biosynthesis and Catabolizing Gene Transcripts in Response to Salt Stress

**FIGURE 6:**
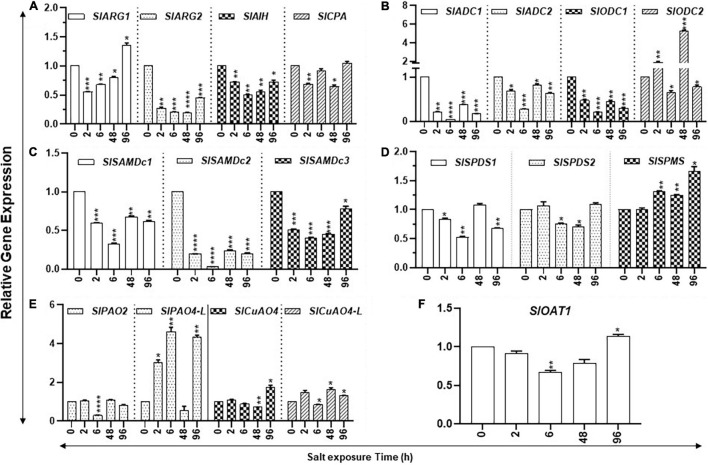
Kinetics of salt stress-induced expression of PA metabolic pathway genes in tomato leaves. Total RNAs were extracted from tomato leaves harvested after salinity stress for 0, 24, 48, and 96 h. The steady state levels of transcripts of various PAs biosynthesis and catabolism genes were determined by qRT-PCR as described in Materials and Methods. Genes included were those of PUT biosynthesis (*SlARG1, 2*, *SlADC1, 2 SlODC1, 2, SlAIH*, and *SlCPA*), SPD, and SPM biosynthesis (*SlSAMDc1, 2, 3, SlSPDS1, 2*, and *SlSPMS*); and catabolism (*SlPAO2* and *SlPAO4-Like; SlCuAO4* and *SlCuAO4-like*) pathways, respectively. *SlOAT1* expression was assessed for the flow of ornithine toward the proline pathway. Mean data points and SE were derived from a minimum of three biological replicates. *SlUB13* and *SlTIP41* were used as reference genes. Other details were the same as in Figure 1. **** indicates significance at *p*-value of <0.00005.

The steady state levels of transcripts of *SlARG1/2*, *SlAIH*, *SlSAMDc3*, and SlOAT1 declined within the first 2 h of salt treatment; after which, their levels increased (Figure 6). Transcripts of *SlCPA*, *SlADC1/2*, *SlODC1*, *SlSAMDc1,2*, and *SlSPDS1* also showed an initial decline, but their patterns thereafter showed mixed patterns (Figure 6). However, *SlSPMS* did not show any initial decline after salt treatment and its transcript levels increased after 6 h and continued up to 96 h of treatment (Figure 6).

Polyamine catabolism pathway genes had specific patterns (Figure 6). For instance, transcript levels of *SlPAO4-like* increased several-fold within 2 h of salt treatment and remained elevated at 6 h but declined at 48 h. By 96 h of the salt treatment, *SlPAO4-like* increased substantially (Figure 6). On the other hand, different patterns of *SlPAO2* under salinity stress were apparent with a sharp decline at 6 h and with only a slight variation at the remaining time points (Figure 6). In regard to catabolic genes, *SlCuAO4* and *SlCuAO4-like* increased at 2 h, declined at 6 h, but then increased significantly at 48 and 96 h of salt treatment.

#### Effect of Salt on Photosynthesis and Carbon Fixation Pathway Gene Expression

**FIGURE 7:**
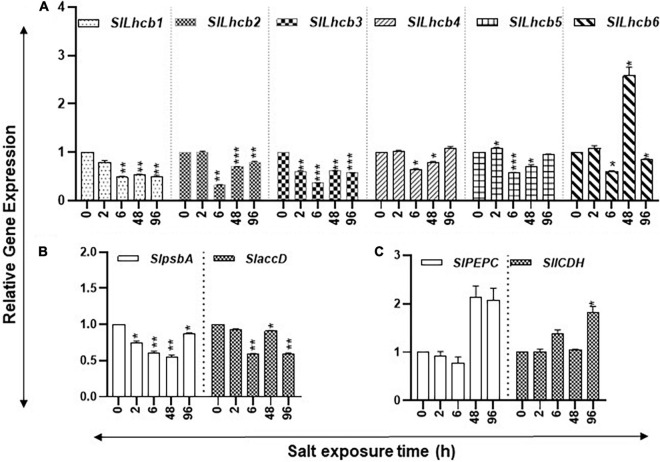
Salt stress and abundance of chlorophyll a/b-binding, photosystem II (PSII), and carbon flow gene transcripts of tomato leaves. Quantitative PCR was used to determine the steady state levels of transcripts of **(A)** nuclear-encoded light-harvesting chlorophyll a/b-binding protein genes (*Lhcb1-6*); **(B)** Plastid-encoded photosystem II (PSII) genes (*psbA* and *accD)*; and **(C)** *phosphoenolpyruvate carboxylase* (*PEPC*) and *isocitrate dehydrogenase* (*ICDH*) genes. Other details were the same as in Figure 1.

The study state levels of carbon assimilation genes during the increasing period of salt stress are shown in Figure 6. The transcript levels of six nuclear-encoded light-harvesting chlorophyll a/b-binding genes (*SlLhcb1*, *SlLhcb2*, *SlLhcb3*, *SlLhcb4*, *SlLhcb5*, and *SlLhcb6*) had variable patterns during the increasing period of salt stress (Figure 7A). *SlLhcb1* gene had a steady decline while *SlLhcb2-5* declined after 6 h, followed by significant increases thereafter, while *Slhcb6* transcript levels increased at 48 h (Figure 7A). Among the two plastid-encoded PSII genes evaluated, *SlpsbA* expression first declined and then increased, whereas *SlaccD* had variable patterns with an increase at 48 h (Figure 7B). Transcript levels of *SlPEPC* increased over twofold after 48- and 96-h salt treatment, while transcript levels of *SlICDH* significantly increased after 96 h (Figure 7C). Statistical differences in salt stress data points are enlisted separately inSupplementary Table S4.

#### Effect of Salt Treatments on Salt-Responsive Genes and Transcription Factors

**FIGURE 8:**
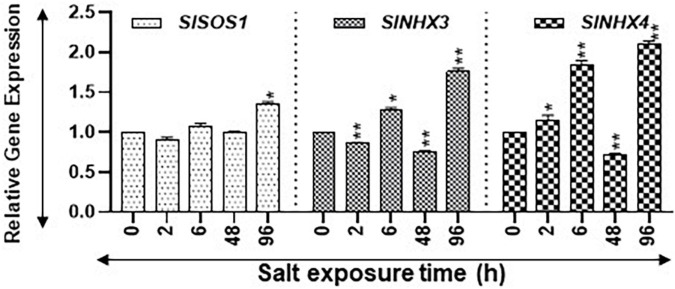
Expression levels of salt stress-related marker genes. Quantitative PCR was used to determine the steady state levels of transcripts of salt-induced marker genes *SlSOS1*, *SlNHX3*, and *SlNHX4*. *SlUBI3* and *SlTIP41* were used as reference genes. Other details are the same as in Figure 1.

Salt Overly Sensitive *SlSOS1* and the Na^+^/H^+^ antiporter (*NHX*) gene family are known to be upregulated during salt stress (Rodríguez-Rosales et al., 2008; Ji et al., 2013; Yang et al., 2017). We quantified the expression of *SlSOS1*, *SlNHX3*, and *SlNHX4* as indicators for salt stress. Transcript levels of *SlSOS1* increased significantly only after 96 h of salt treatment (Figure 8). On the other hand, the transcript levels of *SlNHX3* were significantly higher at 6 h and 96 h but had significant dips at 2 and 48 h of salt treatment (Figure 8). The *SlNHX4* gene transcripts significantly increased at 2, 6, and 96 h after salt treatment, but their significant decline was apparent after 48 h of salt treatment (Figure 8). These patterns suggest that plants were acclimating during early hours of stress and showed signs of seedling revival after 96 h of salt treatment (Figure 8). Statistical differences in salt stress data points are separately listed in Supplementary Table S4.

### Free, Conjugated, and Bound Polyamines to Have Highly Positive Pearson Correlations With One Another Under Drought/Salt Stresses

Pearson correlation coefficient among free, conjugated, and bound forms of PAs was determined during increasing periods of drought and salinity stresses (Supplementary Table S5). Collectively, out of 81 possible combinations among the free, conjugated, and bound forms of PUT, SPD, and SPM for each of the two stresses, 59 and 47 combinations were significant at positive + *r* (>0.4) for both drought and salinity stresses, respectively (Supplementary Table S5). Only four combinations for both drought and salinity were significant with −*r* (< −0.4) (Supplementary Table S5). Strong (negative) −*r* (<−0.8) was observed only for SPM-F, while PUT-B showed −*r* under both drought and salinity stresses (Supplementary Table S5). Under the drought stress, PUT-F had strong + *r* (>0.8) with PUT-C, SPD-F/C/B, and SPD-C/B but not with SPM-F and PUT-B (Supplementary Table S5). A very different pattern was observed under the salinity stress; PUT-F showed strong + *r* only with PUT-C (Supplementary Table S5). SPD-F exhibited + *r* > 0.8 with SPD-C/B and SPM-C/B under drought conditions and with PUT-C/B, SPD-C under the salinity stress, respectively (Supplementary Table S5). Under drought stress, SPD-C and SPM-C had similar patterns since they exhibited + *r* > 0.8 with each other and with PUT-F/C and SPM-B, while SPM-C did not show any correlation (Supplementary Table S5). Under the saline conditions, SPD-C exhibited + *r* > 0.8 with SPD-F/C, PUT-C, and SPM-C, whereas SPM-C had strong + *r* > 0.8 with SPD-C/B and SPM-B (Supplementary Table S5). Taken together, these results reveal coordinately positive regulation of free, conjugated, and bound PAs levels in drought and salinity stresses, most likely to maintain homeostasis.

#### Pearson Correlations Among the Transcript Levels of Polyamine Metabolism Genes With Free, Conjugated and Bound Polyamines Were Mostly Negative Under Drought and Salinity Stresses

Although PA biosynthesis genes had differential levels of transcripts under the drought and saline conditions, their correlations with different forms of PAs were generally negative (Supplementary Table S6). Out of 135 correlation coefficients among the 15 potential PAs biosynthesis genes and 9 forms of PAs (free, conjugated, and bound forms of PUT, SPD, and SPM), correlations for 51 and 40 sets were significantly negative with 33 and 29 combinations with significant + *r* for drought and salinity stress, respectively (Supplementary Table S6). The genes that showed + *r* values > 0.8 with any form of PAs included *ODC2*, *SAMDc1*, and *SPMS* during the drought stress and *ADC1* and *ODC1/2* under the salinity stress, respectively. The PAs biosynthesis genes *SlARG1/2*, *SlAIH1*, *SlCPA*, *SlSAMDc2/3*, *SlSPDS1/2*, *SlSPMS*, and *SlOAT1* showed −*r* under drought. Under salinity stress, *SlARG1/2*, *SlADC1*, *SlAIH1*, *SlCPA*, *SlSAMDc3*, *SlSPDS2*, and *SlSPMS* showed −*r* with several forms of PAs. Collectively, these data indicate that the PA biosynthesis pathway was negatively influenced under both drought and salinity stresses but to a higher extent under drought conditions. Data also indicated that, in some instances, different homologs of genes were affected under the two stresses (Supplementary Table S6). PA catabolism genes were negatively influenced more under drought than salt stress (Supplementary Table S6). Transcript levels of *SlPAO2* and *SlCuAO4-like* were affected by both drought and salt stresses (Supplementary Table S6). This analysis strongly indicates that both drought and salt stresses negatively affect PA metabolism, while drought stress (86 combinations) affects PA metabolism to a higher degree than salinity stress (65 combinations) (Supplementary Table S6).

To evaluate the effects of drought and salinity stresses on the nuclear-encoded light-harvesting chlorophyll a/b-binding, plastid-encoded PSII, carbon flow genes, drought, and salinity stress-associated genes, the Pearson correlation between the transcript levels of selected genes with the levels of free, conjugated, and bound PUT, SPD, and SPM were determined (Supplementary Table S7). Both drought and salinity stress negatively affected these genes in general, but more genes had significantly −*r* under drought (64 combinations) than salt (53 combinations) stress. *SlPEPC* and *SlICDH* had −*r* with most forms of PAs except SPM-F and PUT-B under both drought and salinity stresses. During the drought stress, the *Lhcb1-6* genes had + *r* with several forms of PAs, whereas they showed limited associations with PA forms under salinity stress (Supplementary Table S7). Drought-associated markers *SlRD29A, SlNCED1/2*, and *SlWIRKY57* showed strong −*r* with most forms of PAs but weak correlation with SPM-F and PUT-B. Among the salinity-associated genes, *SlSOS1* showed −*r* with SPD-F, PUT-C, and *SlNHX4* with SPD/SPM-C and SPD/SPM-B. *SlNHX3* did not have a strong association with any form of PAs (Supplementary Table S7).

Coordinate regulation of both drought and salinity stresses with the PAs pathway was examined as well. *SlRD29A*, *SlNCED1/2, SlWIRKY57* exhibited + *r* with *SlARG1/2, SlAIH1, SlODC2*, *SlSAMDc2/3*, *SlSPDS1/2*, *SlOAT1*, *SlPAO2*, *SlPAO4-like*, and *SlCuAO4-like* genes and −*r* with *SAMDC1* genes during the drought stress. *DREB1/2* and *Della* exhibited + *r* with different sets of the PAs pathway that included *ADC1/2, ODC1*, and *SAMDc1* (Supplementary Table S8). *SlNHX3* and *SlNHX4* generally exhibited + *r* with some of the PAs pathway genes. These results suggest that transcription of all drought and salinity stress maker genes tested in this investigation is not coordinately regulated with PAs biosynthesis or catabolism pathways.

#### Coordinate Regulation of Polyamine Biosynthesis and Catabolism Genes

Differential regulation for the PA biosynthesis and catabolism gene transcript levels were observed during drought and salinity stresses. The steady state levels of transcripts for *SlARG1/2*, *SlSAMDc2*, and *SlCuAO4-like* showed coordinate regulation under both drought and salinity stresses (Supplementary Table S8). The genes that showed + *r* under the drought conditions were *SlSAMDc3*, *SlSPDS2*, *SlOAT1*, and *SlPAO2* genes; those under the salinity stress were *SlCPA*, *SlADC1/2*, *SlODC1*, *SlSAMDc1*, and *SlPAO4-like* genes (Supplementary Table S8). *SlODC2* had generally −*r* under drought and +*r* under salinity with some of the genes. In general, among the 284 correlations obtained within the PAs biosynthesis and catabolism genes, 239 exhibited + *r* > 0.8 and 45 exhibited –*r* < −0.8, indicating that coordinate regulation of PAs underlies perception and acclimation during both drought and salinity stresses.

Since photosynthesis plays an essential role in both fresh and dry weight gains leading to total crop yield, we examined coordination among the PAs pathway with a selected set of photosynthesis genes (Figures 3, 7 and Supplementary Table S8). Among the 170 correlations obtained within the PAs metabolic pathway and photosynthesis pathway genes under drought and salinity stresses, 137 showed + *r* > 0.8 and 33 –*r* < −0.8, respectively. However, differential correlation patterns were also seen for several genes during the drought and salinity stresses (Supplementary Table S8). These data indicate that the PA pathway generally contributes positively to the overall expression of selected photosynthesis genes.

#### Coordinate Regulation of Marker and Photosynthesis Genes During Drought and Salinity Stress

Under both drought and salinity stresses, both *SlPEPC* and *SlICDH* showed + *r* > 0.8 among themselves, but *SlpsbA* and *SlaccD* exhibited + *r* > 0.8 only under the drought conditions (Supplementary Table S9). *SlLhcb1-5* showed mostly + *r* > 0.8 among themselves under both drought and salinity stresses. These genes displayed -*r* < −0.8 with *SlPEPC* and *SlICDH* under drought but no correlation under the saline conditions (Supplementary Table S9). Drought-associated marker genes had differential behavior. *SlDREB1/2* and *SlDELLA* were generally + *r* > 0.8 with some photosynthesis genes. *SlRD29A, SlNCED1/2*, and *SlWIRKY57* were coordinately expressed with respect to one another and with + *r* > 0.8 but showed *–r* < −0.8 for *SlLhcb2-5.* Among the salinity-associated marker genes, *SlSOS1* and *SlNHX4* had self-coordination but not with *SlNHX3* transcript levels. *SlNHX3* had + *r* > 0.8 with *SlLhcb3-6*, *SlpsbA*, and *SlaccD* (Supplementary Table S9). These results suggest that factors other than PAs also play roles in the regulation of photosynthesis and marker genes.

### Perchloric Acid Analyses and Differential Segregation of Polyamine Metabolites and the Polyamine Metabolic Pathway, Photosynthetic, and Stress Marker Genes Under Drought and Salinity Stresses

**FIGURE 9:**
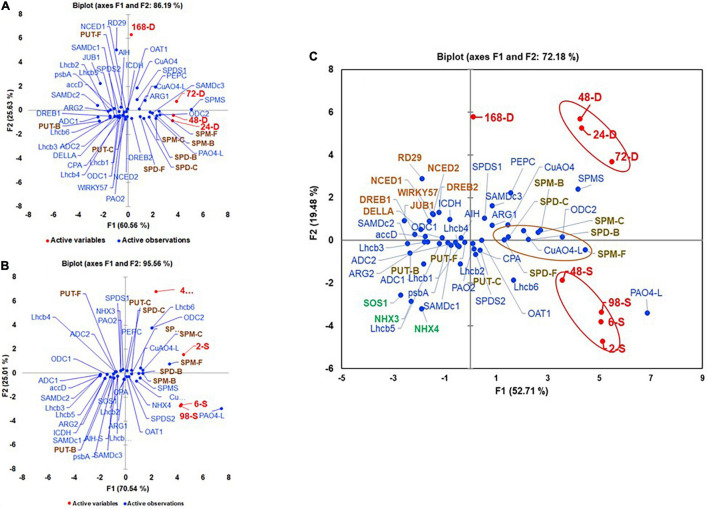
Perchloric Acid analyses of active and variable parameters during **(A)** drought, **(B)** salt stress, and **(C)** their pooled data. The active parameters included leaves exposed to 24, 48, 72, and 168 h of water withholding **(A)** and 2, 6, 48, and 98 h of 200-mM NaCl treatment **(B)** and data from two stresses pooled for each parameter before analysis **(C)**. The variable parameters included levels of free (F), conjugated (C), and bound (B) PUT, SPD, and SPM (PUT-F, SPD-F, SPM-F, PUT-C, SPD-C, SPM-C, PUT-B, SPD-B, and SPM-B). Shown are the transcript levels of PAs biosynthesis and catabolizing enzymes-encoding genes, drought, and salt stress maker genes and photosynthetic machinery-related genes. Other details as stated in the material and method section. Gene abbreviations were the same as in Supplementary Tables S1, S2.

Multivariant analyses of all variables evaluated under the drought and salt stresses are shown in Figures 9A,B. During drought stress, all active variables representing drought samples were present in +PC1 along with SPD-F, SPM-F, SPD-C, SPM-C, SPD-B, and SPM-B but away from the three forms of PUT, suggesting that SPD and SPM but not PUT play roles during perception/acclimation to drought stress. The genes that co-segregated with all the time points of drought included *SlARG1*, *SlSAMDc3*, *SlODC2*, *SlSPDS1*, *SlSPMS*, *SlPAO4-like*, and *SlCuAO4* in +PC1 (Figure 9A). Co-segregation of many SPD/SPM biosynthesis and catabolism genes with drought marker genes *SlDREB2* and *SlPEPC* during drought support the hypothesis that SPD and SPM are significant players in drought stress. Like, under drought stress, all active variables representing salt-treated samples were present in +PC1 (Figure 9B). However, in addition to SPD-F, SPM-F, SPD-C, SPM-C, SPD-B, and SPM-B, PUT-C that segregated with drought stress also co-segregated with salt stress samples, suggesting role(s) of these PAs forms, including PUT-C but not PUT-F and PUT-B in salinity stress (Figure 9B). The genes that showed association with various saline stress samples included *SlODC2*, *SlOAT1*, *SlSPDS2*, *SlSPMS*, *SlPAO4*, *SlPAO4-Like*, *SlCuAO4*, *SlCuAO4-like*, and salinity marker gene *SlNHX4* (Figure 9B). Among the photosynthesis-related genes *SlLhcb 1-6*, only *SlLhcb6* shared + PC1 with salinity samples. Since the first two components, PC1 and PC2 accounted for >86% and >90% variability for drought and saline stresses, respectively, we interpret these results to suggest that the PA biosynthesis and catabolic genes, namely, *ODC2*, *SPMS*, *PAO4-like*, and *CuAO4*, participate in both drought and salinity stresses. However, some of the other genes of biosynthesis and catabolism genes were more associated with drought (*ARG1*, *SAMDc3*, and *SPDS1*) and salinity (*OAT1*, *SPDS2*, *PAO4*, and *CuAO4-like*, respectively).

We also performed combined multivariant analyses of drought and salinity stress datasets to evaluate similarities of responses under the two stresses (Figure 9C). The first two components, PC1 (53.71) and PC2 (19.48) accounted for over 73% of the total variability (Figure 9C). The drought and salinity stress active variables are separated into different quadrants of PCA. All drought active variables (24-D, 48-D, 72-D, and 168-D) were present in +PC1+/+PC2 and that of salt (2-S, 6-S, 48-S, and 96-S) in +PC1/-PC2, indicating that, in spite of the similarities, the two stresses differentially affect PA metabolism in tomato leaves. Although all forms of SPD, SPM, and PUT-C were present close to the *X*-axis of +PC1, SPD-F, SPD-C, and SPD-B had a close association with both the stresses, while SPD-B and SPM-C were closely associated with the drought stress and SPM-F and PUT-C co-segregated with the salinity stress (Figure 9C). These results suggest that, during drought, SPD and SPM homeostasis is tilted toward their bound and conjugated forms, while free SPM and conjugated PUT are tilted more toward salinity stress. As observed in independent drought and salinity analyses, *SlSAMDc3*, *SlODC2*, *SlCuAO4*, and *SlCuAO4-like* were present in +PC1/+PC2, likely indicating that these genes regulate levels of various forms of SPD and SPM during drought stress. Similarly, the association of the salinity stressed samples with *SlSPDS2* and *SlPAO4-like* genes suggests their roles in adaption to salinity stress (Figure 9C). A large number of the PAs pathway and selected photosynthesis genes in −PC1 segregated away from drought and salinity stress samples indicate that their expression is impaired under both stresses and likely responsible for the growth inhibition obtained under these stresses.

### Cytoscape Analyses of Pooled Samples From Drought and Salinity Stress

**FIGURE 10:**
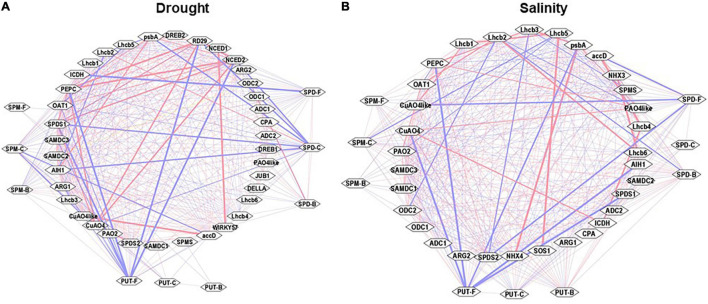
Cytoscape analyses (interactive networks) of different PAs form with the transcript abundances of genes analyzed during drought and salinity stress. Positive (red) and negative (blue) correlations of cellular levels of PUT-F, SPD-F, SPM-F, PUT-C, SPD-C, SPM-C, PUT-B, SPD-B, and SPM-B, and transcript abundance of PAs biosynthesis and catabolizing enzymes genes are shown. Also included in the Cytoscape analysis are the Pearson correlations of selected drought **(A)** and salinity **(B)** stress maker genes and photosynthesis processes and carbon fixation pathway genes. Other details are given in the section “Materials and Methods.”

Cytoscape analyses of the Pearson correlations among observed parameters under the drought and salinity stress were performed to visualize any possible network among these parameters (Figure 10). Free, conjugated, and bound PAs had highly positive Pearson correlations with one another under both stresses. Under drought stress, PUT-F, SPM-C, and SPD-F showed strong negative (−ve) association with transcript levels of *CuAO4-like*, *SlRD29A*, *SlNCED1/2, SlSAMDc1/2*, *SlpsbA*, *SlICDH*, and strong positive (+ve) association with *SlSPDS1/2*, *SlNCED2*, *SlCuAO4*, *SlCuAO4-like*, *SlWIRKY57*, *SlPEPC*, and *SlaacD.* Other observable components had weak positive and negative correlations (Figure 10A). Cytoscape analyses of the Pearson correlations among the observed parameters under the salt stress showed a negative association with *SlSPDS1/2, SlCuOA4, SlPEPC, SlpsbA, SlLhcb1,5*, and *SlaccD* (Figure 10B). Multiple genes were coordinatedly associated among themselves, such as *SlODC2, SlSPMS, SlADC2, SlPAO4-like, SlCuAO4-like, SlNHX3/4, SlpsbA, SlLhcb3,4,6, SlPEPC, SlICDH*, and *SlSOS1*. These results were consistent with the PCA analyses of drought and salinity stress samples (Figures 9A,B). Pooling the data from drought and salinity stresses showed strong negative associations for PUT-F, SPD-C, and SPM-C with *SlRD29A, SlSOS1, SlLhcb2, SlNCED1, SlJUB1*, and *SlNHX4.* However, a very strong positive association was seen among certain pairs of genes that included *SlARG2*, *SlSAMDc2/3*, *SlSPDS1/2*, *SlNHX3/4*, *SlLhcb3*, *SlPEPC*, *SlpsbA*, *SlICDH*, and *SlSOS1*. The correlation coefficients are shown in Supplementary Tables S5–S9.

## Discussion

Polyamines, as plant growth regulators, are known to regulate a myriad of developmental and physiological processes, including longevity in plants under both normal and stress conditions (Gill and Tuteja, 2010; Nambeesan et al., 2010, 2012; Minocha et al., 2014; Mattoo et al., 2015b; Handa et al., 2018; Upadhyay et al., 2020). They have been implicated in developing tolerance or survival under harsh environmental conditions, such as heat, cold, drought, and salinity stress (Kusano et al., 2008; Alcázar et al., 2010; Berberich et al., 2015; Handa et al., 2018; Upadhyay et al., 2020). However, only in limited investigations, the response of plants at different development stages has been analyzed for metabolic changes during time kinetics after induction of drought or salt stress. In this study, we report the complexity as well as unique changes in polyamine metabolic events in tomato leaves during independent drought or salt stress. We particularly demonstrate the effects on the PA biosynthesis and catabolic genes and their impact on the expression of photosynthesis protein-encoding genes in tomato leaves.

Given the fact that drought and salt stresses are far apart in their nature, we found similar changes, by and large, in PA abundance during both stress situations. Even though the levels were not exactly similar, they seemed to mirror each other. The patterns of total PAs and that of free, conjugated, and bound PUT, SPD, and SPM were similar under both types of stresses. However, the cellular levels of each type of PAs were much higher under the salt than drought stress (Figures 1A, 5A). Furthermore, levels of PUT-F decreased similarly in drought and salt stress during later stages, and a similar pattern of increase was apparent in the levels of SPD-F and SPM-F under both the stresses. The data presented suggest that pools of free, conjugated, and bound PAs may be important for the maintenance of PA homeostasis under the two stresses investigated. Moreover, among the three forms of PAs (PUT, SPD, and SPM), both stresses impacted PUT levels negatively, while SPD and SPM were upregulated, suggesting positive roles of SPD and SPM in comparison to the negative role of PUT as previously shown (Handa and Mattoo, 2010; Mattoo et al., 2010).

Interestingly, the levels of total PAs increased with the increasing period of drought (up to 72 h) and salinity (up to 48 h) before declining steeply during prolonged drought (168 h) and salt (96 h) stress (Figures 1, 5). This is in contrast with other reports in which progressive loss of free SPD was observed under water stress in wheat (Marciñska et al., 2013; Hura et al., 2015). The increase in PAs under both stresses was largely due to higher accumulation of SPD with measurable contribution from SPM, while PUT levels gradually declined during the drought and salt stress periods. Moreover, a higher increase in SPD (2,783–3,058 nmol g^–1^ DW) and SPM (739–1,203 nmol g^–1^ DW) was apparent under salt stress than during drought (1,930–21,238 nmol g^–1^ DW SPD and 371- to 605-nmol g^–1^ DW SPM). It is known that higher levels of SPD/SPM contribute to overcoming reactive oxygen species (ROS) by enhancing antioxidative enzymes in plants to overcome salt toxicity (Rider et al., 2007; Chen et al., 2019). The decrease in SPD and SPM levels after a prolonged period of stress likely indicates a loss of cellular viability that results in decreased metabolic activity or oxidation consumption by polyamine oxidase (Cvikrová et al., 2013). However, it remains to be determined if the decreased levels of SPD and SPM are the cause or the effect of loss of cellular vitality after prolonged abiotic stress.

Several investigations have implicated PAs to play significant roles in drought and salinity stress (Kasukabe et al., 2006; Wi et al., 2006; Yamaguchi et al., 2007; He et al., 2008; Alcázar et al., 2010; Liu et al., 2010; Naka et al., 2010; Neily et al., 2011; Chen et al., 2019). Since PAs have been implicated in numerous biological processes, there has been great interest in learning how endogenous PA homeostasis is achieved. Myriad processes are involved in maintaining the optimum cellular levels of various forms of PUT, SPD, and SPM, including their biosynthesis, catabolism, conjugation, and binding to cellular components (Moschou et al., 2008). Interestingly, during heat or cold stress, the change in the levels of free and conjugated PUT, SPD, and SPM is less significant, suggesting that PAs may also utilize different routes to protect plants from cold and high-temperature stress (Upadhyay et al., 2020).

Long-term drought stress has been found to induce structural and functional reorganization of photosystem II of chloroplasts (Giardi et al., 1996). In this context, it is noteworthy that exogenous PAs were found to improve photosynthetic efficiency (Farooq et al., 2009). Similarly, there are some reports that describe the effects of polyamines on the functionality of photosynthetic membrane *in vivo* and *in vitro* in isolated chloroplast from tobacco plants (Ioannidis and Kotzabasis, 2007). Furthermore, it has been shown that saline alkalinity repressed gene expression of psbA gene and protein levels of the D1 protein, and exogenous application of spermidine alleviates expression of psbA gene and D1 protein in salinity-alkalinity stress in tomato seedlings (Hu et al., 2015). As studied here, we found that nuclear light-harvesting chlorophyll a/b-binding protein genes (*SlLhcb1-6*) were downregulated during drought stress and *SlLhcb1-6* were downregulated during salt stress, while during early response to salt, *SlLhcb4*-*6* genes were downregulated (Figures 3A, 7A). The plastid-encoded photosystem II (PSII) protein genes *SlpsbA* (encodes for D1 protein) and *SlaccD* are similarly regulated in both stresses (Figures 3B, 7B). It is also known that Lhcb proteins are downregulated during drought conditions in *Arabidopsis*, but PSII protein downregulation occurs late in response to drought (Chen et al., 2016), which may relate to the fact that PSII is reorganized during drought stress (Giardi et al., 1996). Thus, these data reveal that gene transcription is inhibited earlier during drought stress as compared to the plastid-encoded gene expression for photosynthesis-related genes. Expression analysis of tomato *phosphoenolpyruvate carboxylase* (*SlPEPC*) and *isocitrate dehydrogenase* (*SlICDH*) genes indicated their late activation under both stresses (Figures 3C, 7C). This is indicative of the assertion that markers for the carbon flow in response to drought or salt stress are regulated by a common genetic module. This also suggests that, during acute stress conditions, plants increase carbon fixation and utilization likely by activating the expression of *PEPC* and *ICDH* genes.

Based on the PCA analyses of combined data, SPD-F, SPD-C, and SPD-B cohabit with both drought and salinity stresses. Also, it was clear that SPD-C, SPM-B, and SPM-C segregated with drought stress, while PUT-C and SPM-F segregated with salinity stress. We interpret these results to indicate that differential conjugation of PUT, SPD, and SPM underlies the maintenance of overall PA homeostasis under the two stresses evaluated, and that different conjugated moieties of these PAs play differential roles in stress responses (Figure 9C). Among the PA biosynthesis genes, *SlARG1, SlSPDS1*, and *SlSAMDc3* segregated with drought, while different homologs of *SlSAMDc* and *SlSPDS2* segregated with salt stress, suggesting that specific enzymes, including different isozymes, may regulate drought and salt stress responses. A similar conclusion emerged from the response of catabolic enzymes since *SlCuAO4-like* was associated with drought, while *SlPAO4 was* associated with salt response. Moreover, such responses also emerged from the Cytoscape network analyses of various parameters (Figure 10).

## Data Availability Statement

The original contributions presented in the study are included in the article/Supplementary Material, further inquiries can be directed to the corresponding author/s.

## Author Contributions

RU and AM: conceived and designed the study. RU: bioinformatics and performed the experiments. TF: HPLC/polyamine analysis. RU, AM, AH, and TF: analyzed the final data. AH and AM: facilitated the research, funding grant, and finalized the study. RU, AH, and AM: wrote the manuscript drafts. All authors: contributed to the article and approved the submitted version.

## Conflict of Interest

The authors declare that the research was conducted in the absence of any commercial or financial relationships that could be construed as a potential conflict of interest.

## Publisher’s Note

All claims expressed in this article are solely those of the authors and do not necessarily represent those of their affiliated organizations, or those of the publisher, the editors and the reviewers. Any product that may be evaluated in this article, or claim that may be made by its manufacturer, is not guaranteed or endorsed by the publisher.

## Footnotes

1
solgenomics.net
