# Supplementary material for: Differential Association of Free, Conjugated, and Bound Forms of Polyamines and Transcript Abundance of Their Biosynthetic and Catabolic Genes During Drought/Salinity Stress in Tomato (Solanum lycopersicum L.) Leaves
Source: Front Plant Sci. 2021 Oct 15;12:743568. doi: 10.3389/fpls.2021.743568 (PMC8555666; doi:10.3389/fpls.2021.743568)
Supplement: Supplementary file 1 [file Data_Sheet_1.docx]

**Supplementary Information**

**Differential association of free, conjugated, and bound forms of polyamines and transcript abundance of their biosynthetic and catabolic genes during drought/salinity stress in tomato (*Solanum lycopersicum* L.) leaves**

Rakesh K. Upadhyay^1, 2^, Tahira Fatima^2^, Avtar K. Handa^2^, Autar K. Mattoo^1^*

^1^Sustainable Agricultural Systems Laboratory, United States Department of Agriculture, Agricultural Research Service, Henry A. Wallace Beltsville Agricultural Research Center, Beltsville, MD 20705-2350, USA; ^2^Center of Plant Biology, Department of Horticulture and Landscape Architecture, Purdue University, W. Lafayette, IN 47907, USA

*Correspondence: Autar K. Mattoo ([autar.mattoo@usda.gov](mailto:autar.mattoo@usda.gov))

**Supplementary Tables**

**Table S1:** The sequence identities of PA metabolic pathway genes identified from tomato genome in this study.

**Table S2**: List of genes and their primer sequences used for quantitative real– time PCR (qRT-PCR) analysis in this study.

**Table S3:** Statistically significant differences (p-value) among drought response genes expression studies and polyamines accumulation

**Table S4:** Statistically significant differences (p-value) in salt response time points among genes expression studies and polyamines accumulation

**Table S5**: Correlation among the free, conjugated, and bound PAs

**Table S6**: Correlation of free, conjugated and bound PAs with transcripts levels of PAs biosynthesis and catabolism genes

**Table S7**: Correlation of free, conjugated and bound PAs with photosynthesis, drought and salinity stress related genes

**Table S8**: Pearson correlation among PAs biosynthesis and catabolism genes during drought and salt stress

**Table S9**: Pearson correlation among photosynthesis and drought and salt related genes during drought and salt stress

**Table S1:** The sequence identities of PA metabolic pathway genes identified from tomato genome in this study.

| Process | Polyamine Pathway Enzymes | Gene Name | SGN ID |
| --- | --- | --- | --- |
| Biosynthesis | Arginase (EC 3.5.3.1) | *SlARG1*  *SlARG2* | Solyc01g091160.2.1  Solyc01g091170.2.1 |
|  | Arginine decarboxylase (EC 4.1.1.9) | *SlADC1* | Solyc10g054440.1.1 |
|  |  | *SlADC2* | Solyc01g110440.2.1 |
|  | Ornithine decarboxylase (EC 4.1.1.17) | *SlODC1* | Solyc04g082030.1.1 |
|  |  | *SlODC2* | Solyc03g098300.1.1 |
|  | N-carbamoylputrescine amidohydrolase  (EC 3.5.1.53) | *SlCPA* | Solyc11g068540.1.1 |
|  | Agmatine iminohydrolase /Agmatine deiminase  EC 3.5.3.12) | *SlAIH* | Solyc12g038970.1.1 |
|  | S-adenosylmethionine decarboxylase  (EC 4.1.4.50) | *SlSamDc1* | Solyc05g010420.1.1 |
|  |  | *SlSamDc2* | Solyc02g089610.1.1 |
|  |  | *SlSamDc3* | Solyc01g010050.2.1 |
|  | Spermidine synthase (EC 2.5.1.16) | *SlSPDS1* | Solyc05g005710.2.1 |
|  |  | *SlSPDS2* | Solyc04g026030.2.1 |
|  | Spermine synthase (EC 2.5.1.22) | *SlSPMS* | Solyc08g061970.2.1 |
| Catabolism | Flavin dependent polyamine oxidase  (EC 1.5.3.11) | *SlPAO4-Like* | Solyc02g081390.2.1 |
|  |  | *SlPAO2* | Solyc01g087590.2.1 |
|  | Copper dependent amine oxidase (EC 1.4.3.6) | *SlCuAO* | Solyc08g079430.2.1 |
|  |  | *SlCuAO-Like* | Solyc05g013440.2.1 |

**Table S2**: List of genes and their primer sequences used for quantitative real– time PCR (qRT-PCR) analysis in this study.

| S.No. | Gene Name | SGN ID | Forward Primer (5’-3’) | Reverse Primer (5’-3’) |
| --- | --- | --- | --- | --- |
|  | *SlARG1* | Solyc01g091160 | TACTGCCAAGATATCCAAGTGACC | CCTCCCCAGTAAAGATGAGTACAA |
|  | *SlARG2* | Solyc01g091170 | GTAAGAGAACTTGCTGCCAAGATG | ATCTGCCAGACCTACCTAATGTGA |
|  | *SlADC1* | Solyc10g054440 | CTCGGCGGACTCCATAACC | GCCCAGGGACTGCATAGGT |
|  | *SlADC2* | Solyc01g110440 | GGAGAAGGCGAAGGTGTTG | ATTGGTATTGGTATTGGCATTGG |
|  | *SlODC1* | Solyc04g082030 | TGCGAGCTTTTGCTTCGAAT | GGTAATGCGCCGTATTTTGG |
|  | *SlODC2* | Solyc03g098300 | ACGAAAGAAAGTTGTGAAATACG | CAAGATAGGTAGGTGTGGATAAC |
|  | *SlAIH* | Solyc12g038970 | GGTGCAATTATTACTCCGCAATTT | CAATTTCCCTAGCACCTTCAATTC |
|  | *SlCPA* | Solyc11g068540 | CGAGGGCTATTACTTTTGTCAAGC | CTACTCCAAGCTCCTTTGCAAGAT |
|  | *SlSAMDc1* | Solyc05g010420 | CCTGGTGCTCAATCGTTTCC | TACCGGCAGCAAGCTTTCC |
|  | *SlSAMDc2* | Solyc02g089610 | GCAATGATCGAGAGGGTGTTG | GGCCAGACTCGGTGTAAAGTTC |
|  | *SlSAMDc3* | Solyc01g010050 | CCCGTGGGAGCTTCATCTT | GCTATCAAGGACCGCAACTTCT |
|  | *SlSPDS1* | Solyc05g005710 | GGAGGAGGAGATGGTGGTGTCC | GCAACTCCGTCACCAATGTGGAGAT |
|  | *SlSPDS2* | Solyc04g026030 | AAGGCTCTGTCAACTATGC | GGTGTGGCTATCGTCAAC |
|  | *SlSPMS* | Solyc08g061970 | GATACTGGCATCTTCGTAACTC | GCTTGGTTGTGTTGTAAATG |
|  | *SlPAO2* | Solyc01g087590 | TGATGGAAGGAAGAATGAGAG | CCAGCAGAGGTTCACTTAG |
|  | *SlPAO4-like* | Solyc02g081390 | CCACTTCATATGCTTGCGGTTA | TCGAGGTCACAAGCAAGTCTTC |
|  | *SlCuAO4* | Solyc08g079430 | CGATTTCCCCAATCATCCTTT | CCGCAATTGAATGAACGATTT |
|  | *SlCuAO4-like* | Solyc05g013440 | CAATCGCACTGGGCAGTTAA | CTCCTCAAGAATTTTGCCTCTGA |
|  | *SlOAT1* | Solyc08g048450 | CTTGTGATTGGGAAGAAGTTCG | CAGCCTGAATGCAAAGCATAAC |
|  | *SlTIP41* | Solyc10g049850 | AACCACATTTCAGGCCTTGTCTT | CATGGAGTTTTTGAGTCTTCTGCAT |
|  | *SlUBI3* | Solyc01g056940 | TCGTAAGGAGTGCCCTAATGCTGA | CAATCGCCTCCAGCCTTGTTGTAA |
|  | *SlLHCB1* | Solyc03g005770 | AGTGAAGGAGATCAAGAACGGCA | GTCAGCAATGTGGTCGGCAA |
|  | *SlLHCB2* | Solyc12g006140 | GTGTCACCATGCGACGTACTG | GTCCAGCAGTGTCCCATCCA |
|  | *SlLHCB3* | Solyc07g063600 | CTCTTGAGGTTATCCATGGGAGATG | AGTCCAGCCCACCTTCACTG |
|  | *SlLHCB4* | Solyc09g014520 | GATCCATTGGGCCTTGCTGC | CAAGAACGCAACCATGGCGA |
|  | *SlLHCB5* | Solyc06g063370 | TTGGTGCCAACTGTGGTCCT | AACCTCTGCGACGACAGCAA |
|  | *SlLHCB6* | Solyc01g105050 | TGAGGCTGGACAAGGGAAGAC | ACACTCAACGGCTCTTTCACA |
|  | *SlICDH* | Solyc11g011930 | GGAGAGTTCATCGATGCTGA | TCTGACACCTTAATCCCAAACA |
|  | *SlPEPC* | Solyc07g062530 | ATGAAAGGTATTGCTGCTGGA | TCGAGAAGCTACTAAACAAAGAGG |
|  | *SlpsbA* | NC_007898.3 | ATATCGTAGCCGCTCATGGTTATT | CAGATACCTACTACAGGCCAAGCA |
|  | *SlaccD* | NC_007898.3 | AAGGTTCACAAGCAGCTGAATACT | CGTGGAGCTTAAATAACTCACTCAG |
|  | *SlSOS1* | Solyc01g005020 | TGCGAAAGAAGGCTCAAGTGC | ATGAGGTGCTCATCCTCGCC |
|  | *SlNHX3* | Solyc01g067710 | TCACACTGTGCATCGTTACTGG | TAATGACCACTTGGTTCCGTTG |
|  | *SlNHX4* | Solyc01g098190 | AATGTCCCAGATCTTCCTCACC | ATGAATGCATCGTCAAACTTGC |
|  | *SlNCED1* | Solyc07g056570 | CGCTGAACCATGGCCAAAAGT | TCCCCACCATATTTGTTGTCACC |
|  | *SlNCED2* | Solyc08g016720 | GCCACGACGAGAAGACATGG | CTCTTGAAGGTAGCTTGACAGTAGC |
|  | *SlWIRK57* | Solyc05g012500 | CTATCATCCATCCGGTGTTCAA | TGGTTGACTTGTTTCTGGCAAT |
|  | *SlJUB1* | Solyc05g021090 | TATAGAGGAAGTGCTGGCAAAGGC | CCTACATAGGGTCCAAACTTCAGC |
|  | *SlDREB1* | Solyc06g050520 | CCGTTGCTTCTGGTTTCGGC | ATCGCGGTCCCTTCGTCTTT |
|  | *SlDREB2* | Solyc05g052410 | TGCATTCTGCTGCCTACGATGC | TCGGCATTGTCCAACTGGTATGC |
|  | *SlDELLA* | Solyc11g011260 | TCAAATGCGTTCAAACAAGC | CCACCCCAACATAAGACACC |
|  | *SlRD29A* | Solyc03g025810 | AACAATGAGGAGGCAGGAGTTAGC | GCTTCCGGAACATCGTTCACAC |

**Table S3:** Statistically significant differences (p-value) among drought response genes expression studies and polyamines accumulation

| Category | Genes | Time Points | | | |
| --- | --- | --- | --- | --- | --- |
|  |  | 0h vs 24h | 0h vs 48h | 0h vs 72h | 0h vs 168h |
| PUT Biosynthesis | *SlARG1* | 0.0798^ns^ | 0.5705^ns^ | 0.0637^ns^ | 0.0288* |
|  | *SlARG2* | 0.0001*** | 0.0496* | 0.0023** | 0.0011** |
|  | *SlAIH* | <0.0001**** | 0.1455^ns^ | 0.0010*** | 0.0041** |
|  | *SlCPA* | 0.5090^ns^ | 0.0012** | 0.0570 ^ns^ | 0.0067** |
|  | *SlADC1* | 0.0027** | 0.0030** | <0.0001**** | 0.0002*** |
|  | *SlADC2* | 0.0039** | 0.3051^ns^ | 0.0146* | 0.1854^ns^ |
|  | *SlODC1* | 0.0115* | 0.1205^ns^ | 0.0039** | 0.0034** |
|  | *SlODC2* | 0.1031^ns^ | 0.0027** | 0.0074** | 0.7789^ns^ |
| SPD & SPM Biosynthesis | *SlSAMDc1* | 0.0743^ns^ | 0.0419** | 0.0020** | 0.0018** |
|  | *SlSAMDc2* | 0.0007*** | 0.0001*** | <0.0001**** | 0.0016** |
|  | *SlSAMDc3* | 0.8835^ns^ | 0.8884^ns^ | 0.8871^ns^ | 0.8454^ns^ |
|  | *SlSPDS1* | 0.0099** | 0.1442^ns^ | 0.0764^ns^ | 0.0129* |
|  | *SlSPDS2* | 0.0101* | 0.0102* | 0.0604^ns^ | 0.0199* |
|  | *SlSPMS* | 0.0601^ns^ | 0.0131* | 0.0226* | 0.0153* |
| PA catabolism | *SlPAO2* | 0.0350* | 0.1528^ns^ | 0.0949^ns^ | 0.0122* |
|  | *SlPAO4-like* | 0.2863^ns^ | 0.9195^ns^ | 0.0197^ns^ | 0.1929^ns^ |
|  | *SlCuAO4* | 0.0186* | 0.0230* | 0.0121* | 0.0005*** |
|  | *SlCuAO4-like* | 0.1102^ns^ | 0.0011** | 0.0020** | 0.0028** |
| Towards Proline | *SlOAT1* | 0.9994^ns^ | 0.0613^ns^ | 0.0677^ns^ | 0.0057** |
| Drought Marker genes | *SlDREB1* | 0.0045** | 0.2020^ns^ | 0.0600^ns^ | 0.0471* |
|  | *SlDREB2* | 0.0928^ns^ | 0.0051** | 0.1215^ns^ | 0.1021^ns^ |
|  | *SlDELLA* | 0.0128* | 0.0089** | 0.0017** | 0.0021** |
|  | *SlRD29A* | 0.1346^ns^ | 0.3652^ns^ | 0.9284^ns^ | 0.1434^ns^ |
|  | *SlNCED1* | 0.0174* | 0.4037^ns^ | 0.0300* | 0.0645^ns^ |
|  | *SlNCED2* | 0.1994^ns^ | 0.0421* | <0.0001**** | 0.1782^ns^ |
|  | *SlWIRKY57* | 0.0157* | 0.9447^ns^ | 0.0200* | 0.0091** |
|  | *SlJUB1* | 0.0029** | 0.1187^ns^ | 0.0108* | 0.0178* |
| Light-harvesting chlorophyll a/b-binding protein genes | *SlLHCB1* | 0.0358* | 0.2807^ns^ | 0.0013** | 0.0006*** |
|  | *SlLHCB2* | 0.0074** | 0.0009*** | 0.0049** | <0.0001**** |
|  | *SlLHCB3* | 0.0059** | 0.0028** | 0.0028** | <0.0001**** |
|  | *SlLHCB4* | 0.0713^ns^ | 0.0744^ns^ | 0.3238^ns^ | 0.0132* |
|  | *SlLHCB5* | 0.0168* | 0.0463* | 0.0893^ns^ | 0.0007**** |
|  | *SlLHCB6* | 0.0017** | 0.4645^ns^ | 0.0927^ns^ | 0.0032** |
| PSII protein genes | *SlpsbA* | 0.0009*** | 0.0071** | 0.0023** | 0.0047** |
|  | *SlaccD* | 0.9980^ns^ | 0.0747^ns^ | 0.0075** | 0.0003*** |
| Carbon flow genes | *SlPEPC* | 0.3356^ns^ | 0.3495^ns^ | 0.0074** | 0.0281* |
|  | *SlICDH* | 0.4367^ns^ | 0.0367* | 0.2572^ns^ | 0.0112* |
| Specific PA Forms | PA Types | 0h vs 24h | 0h vs 48h | 0h vs 72h | 0h vs 168h |
| Free | PUT | 0.641^ns^ | 0.9752^ns^ | 0.5248^ns^ | 0.0086** |
|  | SPD | 0.9853^ns^ | 0.2565^ns^ | 0.1316^ns^ | 0.0793^ns^ |
|  | SPM | 0.9842^ns^ | 0.0544^ns^ | 0.0292^*^ | 0.2239^ns^ |
| Conjugated | PUT | 0.8448^ns^ | 0.1672^ns^ | 0.1133^ns^ | 0.0816^ns^ |
|  | SPD | 0.0007*** | 0.0169* | 0.0003*** | 0.0021** |
|  | SPM | 0.0996^ns^ | 0.1605^ns^ | 0.2749^ns^ | 0.6097^ns^ |
| Bound | PUT | 0.9977^ns^ | 0.0112* | 0.0165* | 0.0146* |
|  | SPD | 0.4059^ns^ | 0.9992^ns^ | 0.3615^ns^ | 0.1339^ns^ |
|  | SPM | 0.5724^ns^ | 0.2429^ns^ | 0.3981^ns^ | >0.9999^ns^ |

**Table S4:** Statistically significant differences (p-value) in salt response time points among genes expression studies and polyamines accumulation

| Category | Genes | Time Points | | | |
| --- | --- | --- | --- | --- | --- |
|  |  | 0h vs 2h | 0h vs 6h | 0h vs 48h | 0h vs 96h |
| PUT Biosynthesis | *SlARG1* | 0.0002*** | 0.0022** | 0.0151* | 0.0222* |
|  | *SlARG2* | 0.0016** | 0.0003*** | <0.0001**** | 0.0001*** |
|  | *SlADC1* | 0.0001*** | <0.0001**** | 0.0009*** | 0.0001*** |
|  | *SlADC2* | 0.0160* | 0.0003*** | 0.0395* | 0.0057** |
|  | *SlODC1* | 0.0013** | 0.0001*** | 0.0013** | 0.0004*** |
|  | *SlODC2* | 0.0080** | 0.0102* | 0.0004*** | 0.0225* |
|  | *SlAIH* | 0.0025** | 0.0028** | 0.0065** | 0.0271* |
|  | *SlCPA* | 0.0024** | 0.2380^ns^ | 0.0117* | 0.6251^ns^ |
| SPD & SPM Biosynthesis | *SlSAMDc1* | 0.0003*** | 0.0003*** | 0.0045** | 0.0058** |
|  | *SlSAMDc2* | <0.0001**** | <0.0001**** | 0.0006*** | 0.0003*** |
|  | *SlSAMDc3* | 0.0008*** | 0.0002*** | 0.0009*** | 0.0485* |
|  | *SlSPDS1* | 0.0287* | 0.0017** | 0.1245^ns^ | 0.0049** |
|  | *SlSPDS2* | 0.7296^ns^ | 0.0116* | 0.0173* | 0.1403^ns^ |
|  | *SlSPMS* | 0.9988^ns^ | 0.0097** | 0.0061** | 0.0300* |
| PA catabolism | *SlPAO2* | 0.6548^ns^ | <0.0001**** | 0.4926^ns^ | 0.0632^ns^ |
|  | *SlPAO4-like* | 0.0126* | 0.0077** | 0.3297^ns^ | 0.0025** |
|  | *SlCuAO4* | 0.4110^ns^ | 0.1981^ns^ | 0.0064** | 0.0333* |
|  | *SlCuAO4-like* | 0.0820^ns^ | 0.0380* | 0.0399* | 0.0159* |
| Towards Proline | *SlOAT1* | 0.2474^ns^ | 0.0094** | 0.1054^ns^ | 0.0476* |
| Salt Marker genes | *SlSOS1* | 0.1895^ns^ | 0.3369^ns^ | 0.9993^ns^ | 0.0156* |
|  | *SlNHX3* | 0.0019** | 0.0247* | 0.0069** | 0.0055** |
|  | *SlNHX4* | 0.2103^ns^ | 0.0111* | 0.0065** | 0.0015** |
| Light-harvesting chlorophyll a/b-binding protein genes | *SlLHCB1* | 0.0627^ns^ | 0.0035** | 0.0034** | 0.0033** |
|  | *SlLHCB2* | 0.9993^ns^ | 0.0011** | 0.0003*** | 0.0077** |
|  | *SlLHCB3* | 0.0015** | 0.0003*** | 0.0010** | 0.0003*** |
|  | *SlLHCB4* | 0.7382^ns^ | 0.0172* | 0.0111* | 0.4528^ns^ |
|  | *SlLHCB5* | 0.0438* | 0.0007*** | 0.0344* | 0.1553^ns^ |
|  | *SlLHCB6* | 0.5365^ns^ | 0.0024* | 0.0265* | 0.0140* |
| PSII protein genes | *SlpsbA* | 0.0204* | 0.0054** | 0.0075** | 0.0201* |
|  | *SlaccD* | 0.0827^ns^ | 0.0030** | 0.0236* | 0.0030** |
| Carbon flow genes | *SlICDH* | 0.9981^ns^ | 0.0947^ns^ | 0.1587^ns^ | 0.0346* |
|  | *SlPEPC* | 0.7437^ns^ | 0.4258^ns^ | 0.1400^ns^ | 0.0561^ns^ |
| Specific PA Forms | PA Types | 0h vs 2h | 0h vs 6h | 0h vs 48h | 0h vs 96h |
| Free | PUT | 0.2358^ns^ | 0.3467^ns^ | 0.7611^ns^ | 0.0279* |
|  | SPD | 0.8954^ns^ | 0.9443^ns^ | >0.9999^ns^ | 0.5160^ns^ |
|  | SPM | 0.2201^ns^ | 0.2564^ns^ | 0.0869^ns^ | 0.0314* |
| Conjugated | PUT | 0.9642^ns^ | 0.9916^ns^ | 0.9990^ns^ | 0.4794^ns^ |
|  | SPD | 0.6653^ns^ | 0.2581^ns^ | 0.0875^ns^ | 0.0155* |
|  | SPM | 0.1719^ns^ | 0.1237^ns^ | 0.0841^ns^ | 0.9568^ns^ |
| Bound | PUT | 0.1878^ns^ | 0.2442^ns^ | 0.0553^ns^ | 0.0548^ns^ |
|  | SPD | 0.8705^ns^ | 0.2310^ns^ | 0.1859^ns^ | 0.4695^ns^ |
|  | SPM | 0.2444^ns^ | 0.1192^ns^ | 0.0950^ns^ | 0.2801^ns^ |

**Table S5: Correlations among the free, conjugated and bound PAs**

Tabulated *r* values (DF 14) > 0.5, > 0.62 and > 0.72 are significant

at p-value < 0.05, <0.01 and < 0.001, respectively

**Table S6: Correlations of free, conjugated and bound PAs with transcripts levels of PAs biosynthesis and catabolism genes**

Tabulated *r* values (DF 14) > 0.5, > 0.62 and > 0.72 are significant

at p-value < 0.05, <0.01 and < 0.001, respectively

**Table S7: Correlations of free, conjugated and bound PAs with Photosynthesis, drought and salinity stress related genes**

Tabulated *r* values (DF 14) > 0.5, > 0.62 and > 0.72 are significant

at p-value < 0.05, <0.01 and < 0.001, respectively

**Table S8. Pearson correlation among PAs biosynthesis and catabolism genes during drought and salt stress**

Tabulated *r* values (DF 14) > 0.5, > 0.62 and > 0.72 are significant

at p-value < 0.05, <0.01 and < 0.001, respectively

**Table S9. Pearson correlation among photosynthesis and drought and salt related genes during drought and salt stress**

Tabulated *r* values (DF 14) > 0.5, > 0.62 and > 0.72 are significant

at p-value < 0.05, <0.01 and < 0.001, respectively
